# Implications of Soil Pollution with Diesel Oil and BP Petroleum with ACTIVE Technology for Soil Health

**DOI:** 10.3390/ijerph16142474

**Published:** 2019-07-11

**Authors:** Agata Borowik, Jadwiga Wyszkowska, Mirosław Kucharski, Jan Kucharski

**Affiliations:** Department of Microbiology, University of Warmia and Mazury in Olsztyn, 10-727 Olsztyn, Poland

**Keywords:** bacteria metagenomics, enzymatic activity, grass resistance, PAHs degradation

## Abstract

Grass *Elymus elongatus* has a potential in phytoremediation and was used in this study in a potted experiment, which was performed to determine the effect of polluting soil (Eutric Cambisol) with diesel oil (DO) and unleaded petroleum (P) on the diversity of soil microorganisms, activity of soil enzymes, physicochemical properties of soil, and on the resistance of *Elymus elongatus* to DO and P, which altogether allowed evaluating soil health. Both petroleum products were administered in doses of 0 and 7 cm^3^ kg^−1^ soil d.m. Vegetation of *Elymus elongatus* spanned for 105 days. Grasses were harvested three times, i.e., on day 45, 75, and 105 of the experiment. The study results demonstrated a stronger toxic effect of DO than of P on the growth and development of *Elymus elongatus*. Diesel oil caused greater changes in soil microbiome compared to unleaded petroleum. This hypothesis was additionally confirmed by Shannon and Simpson indices computed based on operational taxonomic unit (OTU) abundance, whose values were the lowest in the DO-polluted soil. Soil pollution with DO reduced the counts of all bacterial taxa and stimulated the activity of soil enzymes, whereas soil pollution with P diminished the diversity of bacteria only at the phylum, class, order, and family levels, but significantly suppressed the enzymatic activity. More polycyclic aromatic hydrocarbons (PAHs) were degraded in the soil polluted with P compared to DO, which may be attributed to the stimulating effect of *Elymus elongatus* on this process, as it grew better in the soil polluted with P than in that polluted with DO.

## 1. Introduction

Petroleum hydrocarbons represent the largest group of organic pollutants [1,2]. They are highly resistant to biodegradation, capable of accumulating in plants as well as in human and animal bodies [3,4], and exhibit carcinogenic and neurotoxic properties [3,5]. 

The growing pollution of the natural environment urges the search for effective remediation methods. One of these is phytoremediation, which makes use of the natural capabilities of plants for growth and development on polluted areas [5,6,7,8]. 

Interactions between plants, soil microbiome, and soil pollution with petroleum substances are complex in character and require a variety of analyses, while little is known about the impact of petroleum products on the biodiversity in agricultural ecosystems. Establishing links between microbiological diversity and soil functions is not an easy task [9,10]. Changes in the stability of soils and their ecological processes are affected by chemical pollutants [3,6,7,11], climate changes [12] or plant root secretions [13]. According to Haney et al. [14], plants provide substrates to microorganisms and, in exchange, rhizospheric microorganisms provide nutrients and phytohormones, increase immunity, and inhibit the growth of phytopathogens. 

Phytoremediation is deemed not only a promising technology for the treatment of polluted soils, but also one of the most cost-effective ones in this respect [15,16,17]. It includes the following methods: phytoextraction, phytodegradation, phytotransformation, rhizofiltration, phytostabilization, and phytovolatilization. Their use was reported to diminish the bioavailability of pollutants, both the organic and inorganic ones, through their immobilization or their binding with soil matter, their accumulation in biomass, degradation, or transformation by both roots and aerial parts of plants. Alternatively, some substances were emitted to the atmosphere as a result of methylation [18]. Attempts of phytoremediation of soils polluted with petroleum products have been undertaken with the following herbaceous plants: *Cyperus rotundus*, *Chloris babata*, *Pasparlum vaginatum*, *Paspalum scrobiculatum*, *Euragrostis atrovilens* [16], *Cynodon dactylon*, *Digitaria sanguinalis,* and *Cyperus orthostachyus* [19]; also with: *Acorus calamus* [20], *Chromolaena odorata*, *Aspilla africana*, *Bryophylum pinnatum* [16], and *Zea mays* [21]; and with trees: *Spondias mombim* [16], *Betula pendula* [22], and *Salix* varieties [23]. 

Plants used in the phytoremediation process should be characterized by an intensive growth in polluted soil, capability for pollutants accumulation (phytomining) [24], and a high calorific value. One of the plants displaying a large phytoremediating potential is a perennial energy grass *Elymus elongatus* (*Agropyrum elengatum*) of Bamar variety. Its common name is tall wheatgrass. *Elymus* grows in abundant plant-colonizing meadows, areas alongside river banks, and mountainsides—especially at high altitudes ranging from 1000 to 4000 m, on the Qinghai-Tibetan plateau in western and northern China [25,26,27], regions of the Tibetan plateau, and interior Mongolian plateau [28]. This species grows in the moderate, subtropical, and sub-Alp climates on acidic, salinated, and alkaline soils, and is highly resistant to diseases and to biotic and abiotic stress. Interestingly, it is closely related with a few important cereal species, like wheat, barley, and rye [27,28]. Its dry matter yield ranges from 13 to 25 Mg ha^−1^ [29]. The high resistance of tall wheatgrass to the abiotic stress has made it utile for the remediation of soil polluted with petroleum products. 

Considering the above, this study aimed to determine the feasibility of using *Elymus elongatus* for restoring the biological homeostasis of soil polluted with BP (British Petroleum Company) diesel oil and unleaded petroleum with Active technology. This evaluation was made based on determinations of grass biomass, activity of soil enzymes, genetic diversity of bacteria, and PAHs degradation.

## 2. Materials and Methods 

### 2.1. Soil 

This study was conducted with samples of soil, which are classified as Eutric Cambisol. Soil samples were collected from the area located in north-east Poland (53.7161 N, 20.4167 E). This region is characterized by the climate of the moderate warm transient zone, determined by natural conditions, including lakes and forests located in the vicinity. According to the Institute of Meteorology and Water Management State Research Institute (IMGW) in Poland, the average temperature in this region in the period of June–September was ca. 17 ± 2 °C, ranging from min. 7 °C to max. 30 °C. Insolation ranged from 280 h in July to 130 h in September, and the mean total precipitation was at ca. 152.50 mm. 

The soil was composed of (in 1 kg d.m.): 74.93 g of sand fraction, 22.85 g of dust fraction, and 2.22 g of clay fraction. It contained (in 1 kg d.m.): 0.62 g of total nitrogen (N_tot_), 9.30 g of organic carbon (C_org_), 93.68 mg of phosphorus (P), 141.10 mg of available potassium (K^+^), 42.0 mg of available magnesium (Mg^2+^), and the following exchangeable cations: 156.0 mg K^+^, 623.5 Ca^2+^, 59.5 Mg^2+^, and Na^+^ 4.00. In addition (in 1 kg d.m.), its hydrolytic activity (Hh) reached 11.4 mM (H^+^); sum of its total exchangeable base cation (S)—49.0 mM(+), and exchangeable capacity of the sorption complex (T)—60.4 mmol(+). Soil saturation with cations (V) was at 81.1%, and its pH in 1 mol KCl dm^−3^ was 6.7.

### 2.2. Plant 

Soils polluted with diesel oil and petroleum were phytoremediated using perennial grass *Elymus elongatus* (*Agropyrum elongatum*) var. Bamar. This grass species is adapted to the Polish climate. The first seeds of rare wild forms of var. Bamar originated from salinated, arid soil from the borderline between Asia and south-east Europe. The study conducted by Martyniak et al. [29] demonstrated that *Elymus elongatus* seeds are highly capable of sprouting even on sandy, impoverished, and degraded soils. This plant is characterized by intense tillering, the production of a high number of vegetative shoots reaching up to 2 m in height, and a deep root system. Compared to other energy crops, the biomass of *Elymus elongatus* var. Bamar has a high calorific value. After incineration, its dry matter is characterized by a low ash content. 

### 2.3. Petroleum Products 

The study was conducted with diesel oil and petroleum purchased at a BP gas station. The BP diesel oil with Active technology (DO) and BP 98 unleaded petroleum with Active technology (P) are fuels that remove dirt from the engine and prevent power loss, increased consumption of fuel, and wear of engine elements. According to information provided by the fuel distributor, both petroleum substances meet criteria set in EC 1907/2006 (REACH). The BP diesel oil is an over 90% mixture of hydrocarbons C_10_–C_28_, likely to contain fatty acid methyl esters (FAME). In turn, the BP 98 petroleum is a mixture of volatile hydrocarbons; it contains, paraffins, napthenes, olephins, and aromatic compounds with C_4_–C_12_. The BP fuels contain also small amounts of enriching substances and multiple molecules that capture dirt, thereby protecting the engine and aiding its work [30].

### 2.4. Experimental Procedure 

To avoid soil pollution in its natural ecosystem, the study was conducted under controlled conditions of an ex situ experiment. The soil was sampled from a depth of 0–20 cm at the Didactic-Experimental Station of the University of Warmia and Mazury in Olsztyn (north-eastern Poland) and transported to a greenhouse, wherein it was mixed and sieved through a screen with a mesh size of 1 cm, and then used to establish the pot experiment. The experiment was performed in Kick-Brauckman pots in 4 replications, for 105 days. The experimental objects were: 1) unpolluted soil sown with *Elymus elongatus*, 2) soil sown with *Elymus elongatus* and polluted with 7 cm^3^ of diesel oil BP with Active technology (DO) kg^−1^ of soil d.m., and 3) soil sown with *Elymus elongatus* and polluted with 7 cm^3^ of BP 98 unleaded petroleum with Active technology (P) kg^−1^ of soil d.m. Soil samples (9 kg) were carefully mixed with 720 mg of N in the form of CO(NH_2_)_2_, 180 mg of P in the form of KH_2_PO_4_, 360 mg of K in the form of KCl and KH_2_PO_4_, and with 90 mg of Mg in the form of MgSO_4_ · 7H_2_O. Afterwards, the soil was mixed with DO or P in respective experimental series and packed into polyethylene pots with the volume of 7.5 dm^3^. After 1 week, 24 seeds *Elymus elongatus* were sown to each pot. Assumptions of the experiment performed under controlled conditions allowed monitoring soil humidity that was kept at the level of 60% using distilled water. Day time ranged from 13 h 3 min to 16 h 31 min. The average air temperature was 15.6 °C and air humidity was 76.5%. Grasses were harvested 3 times, i.e., on day 45, 75, and 105 of the experiment. 

### 2.5. Methodology of Microbiological Analyses 

#### 2.5.1. Bacterial and Fungal Counts 

Once the experiment had been terminated, soil samples from each pot were determined for counts of organotrophs (Org), Actinobacteria (Act), and fungi (Fun) with the serial dilutions method acc. to the procedure described in the work by Borowik et al. [10]. Soil samples (10 g) were weighed to a sterile physiological saline solution (90 cm^3^ of 0.85% NaCl) and shaken for 30 min at 120 rpm. The tests were carried out in four repetitions. The composition of microbiological media were as follows: for the organotrophic bacteria (Bunt and Rovira medium): agar medium (peptone 1.0 g, yeast extract 1.0 g, (NH_4_)_2_SO_4_ 0.5 g, CaCl_2_, K_2_HPO_4_ 0.4 g, MgCl_2_ 0,2 g, MgSO_4_ 7H_2_O 0.5 g, Mo salt 0.03 g, FeCl_2_ 0.01 g, agar 20.0 g, soil extract 250 cm^3^, distilled water 750 cm^3^, pH 6.6–7.0; for Actinobacteria (Parkinson medium): soluble starch 10.0 g; casein 0.3 g; KNO_3_ 2.0 g; NaCl 2.0 g; K_2_HPO_4_ 2.0 g; MgSO_4_⋅7H_2_O 0.05 g; CaCO_3_ 0.02 g; FeSO_4_ 0.01 g; agar 20.0 g; H_2_O 1 dm^3^; 50 cm^3^ aqueous solution of nystatin 0.05%; 50 cm^3^ aqueous solution of actidione 0.05%; pH 7.0; and for fungi (Martin medium): peptone 5 g; K_2_HPO_4_ 1.0 g; glucose 10 g; MgSO_4_⋅7H_2_O 0.5 g; agar 20.0 g; H_2_O 1 dm^3^; 3.3 cm^3^ aqueous solution of bengal rose 1%; 25 cm^3^ aqueous solution of aureomycin 0.01%; pH 5.9. Microorganisms were cultured on Petri dishes at a temperature of 28 °C, within a period of 10 days. 

The number of colony-forming units (cfu) was established using a colony counter. Microbial counts determined for 10 subsequent days were used to compute the colony development index (CD) and the ecophysiological diversity index (EP) of microorganisms acc. to De Leij et al. [31] based on the following formulas:

CD = [N1/1 + N2/2 + N3/3….. N10/10] · 100(1)

where: N1, N2, N3, …, N10—the sum of ratios of the number of colonies of microorganisms identified in particular days (1, 2, 3, …, 10) to the total number of colonies identified throughout the study period, and:

EP = −Σ(pi·log10 pi)(2)

where: pi—the ratio of the number of colonies of microorganisms identified in particular days to the total number of colonies identified throughout the study period.

#### 2.5.2. DNA Extraction and Bioinformatic Analysis of Specific Bacterial Taxa

DNA was extracted from 1 g of soil with a “Genomic Mini AX Soil+” kit. The presence of bacterial DNA in the soil samples was confirmed with the Real-Time PCR, which was performed in an Mx3000P thermocycler (Stratagene), using an SYBR Green dye (A&A Biotechnology) as a fluorochrome. The reaction was conducted with 1055F primer (5′-ATGGCTGTCGTCAGCT-3′) and 1392R primer (5’-ACGGGCGGTGTGTAC-3’) which amplify the fragment of bacterial 16SrDNA gene. Sequencing was performed by an external company (Genomed S.A. Warsaw, Poland) on an MiSeq sequencer in the paired-end (PE) technology, 2 x 250 bp, using v2 Illumina kit. The metagenomic analysis of bacteria and archeons was carried out based on hypervariable region V3-V4 of the 16S rRNA gene. The bioinformatic analysis, enabling classification of the read out to the species level, was conducted using QIIME package based on GreenGenes v13_8 database of reference sequences. 

### 2.6. Methodology of Biochemical Analyses

Once the experiment had been completed, soil samples from each pot were determined for the activity of seven soil enzymes, including two from the class of oxidoreductases: dehydrogenases (EC 1.1) and catalase (EC 1.11.1.6), and five classified to hydrolases: urease (EC 3.5.1.5), acid phosphatase (EC 3.1.3.2), alkaline phosphatase (EC 3.1.3.1), arylsulfatase (EC 3.1.6.1), and β-glucosidase (EC 3.2.1.21). The activity of dehydrogenases was determined acc. to Öhlinger [32], that of catalase with the titration method using potassium permanganate [33], whereas activities of the other enzymes acc. to Alef and Nannpieri [34]. Substrates used for enzymatic activity determinations included aqueous solutions of the following chemical compounds: 2,3,5-triphenyl tetrazolium chloride (TTC) for dehydrogenases, urea for urease, disodium 4-nitrophenyl phosphate hexahydrate (PNP) for phosphatases, potassium-4-nitrophenylsulfate (PNS) for arylsulfatase, and 4-nitrophenyl-β-D-glucopyranoside (PNG) for β-glucosidase. Activities of all enzymes except for catalase were determined using a Perkin-Elmer Lambda 25 spectrophotometer (Massachusets, USA). They were converted into the amount of product obtained within 1 h by 1 kg soil d.m. and expressed in the following units: dehydrogenases—μmol TFF (tri-phenylformazan); catalase—mol O_2_; acid phosphatase, alkaline phosphatase, arylsulfatase, and β-glucosidase—mmol PN (p-nitrophenol); and urease—mmol N-NH_4_. 

### 2.7. Methodology of Chemical and Physiochemical Analyses of Soil 

The fraction composition of soil was determined with a laser meter, the pH of soil in 1 mol KCl dm^−3^ [35], hydrolytic acidity (HAS) and sum of exchangeable base cations (EBC) acc. to Carter and Gregorich [36], organic carbon acc. to Tiurin [37], total nitrogen with the Kjeldahl method [38], available phosphorus and potassium with the Egner et al. method [39], and magnesium with atomic absorption spectrometry (AAS) [40]. Exchangeable cations: K^+^, Ca^2+^, Mg^2+^, and Na^+^ were determined following the procedure described in PN-EN ISO 11260 [41].

Both before the experiment had been established and after its completion, soil samples were determined for the contents of: benzines (C_6_–C_12_), mineral oils (C_12_–C_35_), volatile aromatic hydrocarbons (BETX), and PAHs with 2 rings (naphthalene), 3 rings (anthracene), 4 rings (chrysene, benzo(a)anthracene), 5 rings (dibenz(ah)anthracene, benzo(a)pyrene, benzo(b)fluoranthene, benzo(k)fluoranthene), and 6 rings (benzo(ghi)perylene, indo(123-cd)pyrene), and for ∑10 PAHs. Soil samples collected from pots for GC analyses were sieved through a screen with mesh size of 2 mm, then immediately packed to special containers by Wessling company, and delivered for analyses on the same day. Contents of PAHs were determined at Wessling (Kraków, Poland) on a gas chromatograph with an Agilent 7890A-5975C mass spectrometer equipped in EI/CI ion source acc. to the following standards: ISO 18287 [42], EN ISO 16703 [43], and EN ISO 22155 [44]. A methanolic extract of soil samples was prepared for determinations of volatile compounds (benzines, BTEX). A weighted portion of soil with the addition of methanol was shaken, sonicated, and centrifuged. The resultant methanolic extract was transferred to a headspace type vial that was incubated in a thermostatic mixer. Once the equilibrium was settled between the liquid and gaseous phases, the amount of the gaseous phase was determined and the phase was transferred to an injector of a gas chromatograph. The quantitative and qualitative composition of the sample was determined using a mass detector based on the internal standard added.

To determine the contents of mineral oils (C_12_–C_35_) and polycyclic aromatic hydrocarbons (PAHs), anhydrous sodium sulfate (VI) was added to weighted portions of soil, then the sample was homogenized with acetone and shaken in a horizontal shaker. Afterwards, it was double extracted with hexane in an ultrasound bath. The resultant extract was dried with anhydrous sodium sulfate (VI), transferred to a test tube, and concentrated to the volume of 5 cm^3^ using a concentrator at a temperature of 40 °C. Afterwards, 1 cm^3^ of the extract was collected and filtered through a column filled with 2 g of silica gel. Fractions of aliphatic hydrocarbons were eluted with 9 cm^3^ of hexane (for determinations of C_12_–C_36_), whereas PAHs were eluted with 18 cm^3^ of dichloromethane. The collected eluates were concentrated to the volume of 1 cm^3^ and analyzed.

### 2.8. Statistical Analysis

Results were processed in the Statistica 13.1 package (StatSoft, Tulsa, OK, USA) [45], using Principal Component Analysis (PCA). Homogenous groups were calculated with the Tukey’s test, at *P* = 0.05. In addition, the index of plants and enzymes resistance (RS) to effects of petroleum products and the index of plant adaptation (RL) to pollution were calculated using formulas proposed by Orwin and Wardle [46]. Relative abundance was visualized by means of STAMP 2.1.3 software, using a two-sided test of statistical hypotheses: G-test (w/Yates’) + Fisher’s, with the method of intervals confidence Asymptotic with CC [47]. In turn, the Circos 0.68 package [48] was used to present genomic data in the circular system. The visualization of relative abundance was performed only with sequences with a contribution that exceeded 1%. In addition, to determine bacterial diversity, all metagenomic data were analyzed with the use of Shannon–Wiener (H) and Simpson (D) indices at the level of each taxonomic group.

## 3. Results

### 3.1. Counts and Diversity of Microorganisms in the Soil 

**Figure 1:**
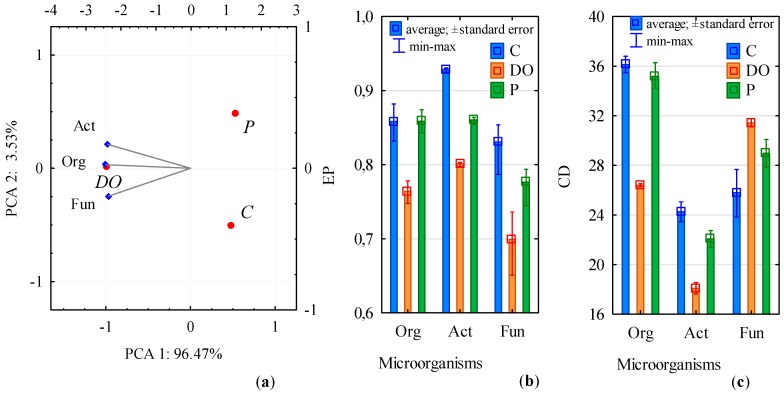
Microbiological properties of uncontaminated soil (C), soil contaminated with diesel oil (DO) and unleaded petroleum (P); (**a**) count of soil microorganism presented by the Principal Component Analysis (PCA) method; (**b**) physiological diversity index of microorganisms (EP); (**c**) colony development index (CD). Homogeneous groups denoted with letters (a, b, c) were calculated separately for each of microorganism. Org—organotrophic bacteria, Act—Actinobacteria, Fun—fungi.

The proliferation of all microorganisms in the soil was significantly stimulated by the BP diesel oil with Active technology (DO), whereas the BP 98 unleaded petroleum with Active technology (P) caused no changes in the population numbers of organotrophic bacteria and fungi (Figure 1a), while it enhanced the proliferation of actinobacteria. Despite the positive effect of DO on the proliferation of organotrophic bacteria, it contributed to their ecophysiological diversity index (EP) decrease from 0.85 to 0.76 (Figure 1b). Values of this index decreased also in the case of Actinobacteria and fungi. In the series with soil pollution with P, its value did not change significantly in the case of organotrophic bacteria and fungi but decreased significantly in the case of Actinobacteria. In turn, values of the colony development (CD) index of microorganisms indicated the slowest development of actinobacteria and the fastest development of organotrophic bacteria in the soil (Figure 1c). Effects of the petroleum products on the development of microorganism colonies varied. DO inhibited the development of organotrophic bacteria and actinobacteria colonies and enhanced that of fungi. Petroleum decreased the CD value only for Actinobacteria. 

**Figure 2:**
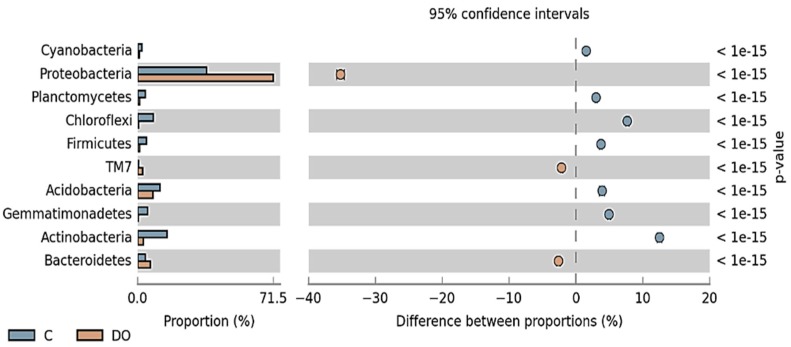

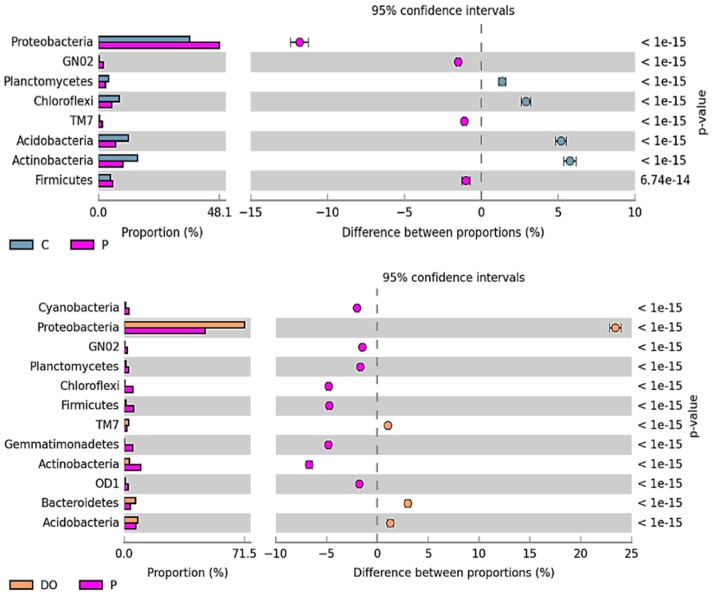
The relative abundance of dominant phylum of bacteria in the soil with the difference between proportions ≥1%. C—uncontaminated soil, DO—soil contaminated with diesel oil, P—soil contaminated with unleaded petroleum.

In all soil samples, the prevailing *Phylum* was *Proteobacteria* (Figure 2). They accounted for 36.2% in the control soil (unpolluted), for 48.1% in the soil polluted with P, and for 71.5% in the soil exposed to DO contamination. The OTU number of *Proteobacteria* in the DO-polluted soil was higher by 35.3%, and in the P-polluted soil by 11.9%. *Actinobacteria* in the control soil and P-polluted soil (accounting for 15.6 and 9.8%) and *Acidobacteria* in the DO-polluted soil (8.0%) were the second highest after the *Phylum Proteobacteria*. The pollution of soil with petroleum products elicited significant changes in its microbiome. The OTU number of *Actinobacteria* in the control soil was higher by 12.5% than in the soil polluted with DO and by 5.8% than in the soil polluted with P. 

**Figure 3:**
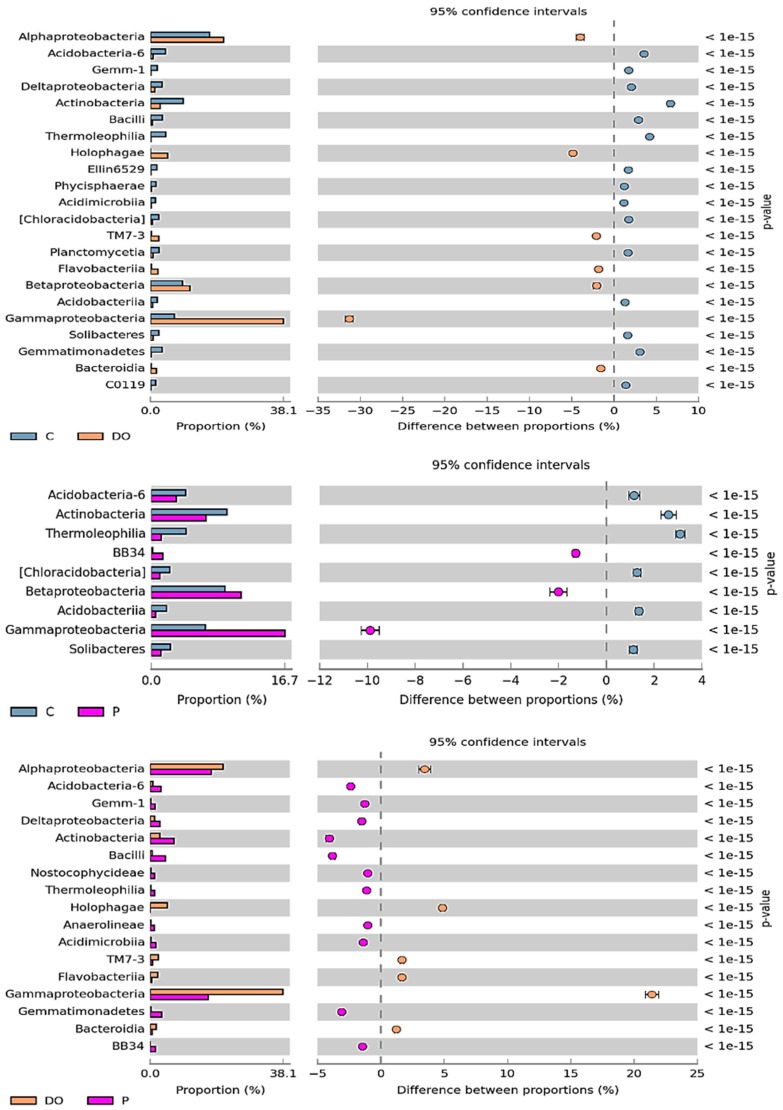
The relative abundance of dominant classes of bacteria in the soil with difference between proportions ≥1%. C—uncontaminated soil, DO—soil contaminated with diesel oil, P—soil contaminated with unleaded petroleum.

The greatest changes were caused by the petroleum products in the class *Gammaproteobacteria* (Figure 3). Compared to the control soil, an increase in OTU number induced by DO reached 31.3%, while when induced by P it reached 9.9%. DO had a negative effect on *Actinobacteria* class bacteria (OTU number decrease by 6.7%), whereas P on *Thermoleophilia* (OTU number decrease by 3.1%). Differences were also found between DO and P in their effects on *Gammaproteobacteria*, because OTU number increased upon soil pollution with DO by 21.4% compared to soil pollution with P. Differences in the other analyzed classes were considerably smaller and ranged from −4.1% (*Actinobacteria*) to 4.9% (*Holophagae*). 

**Figure 4:**
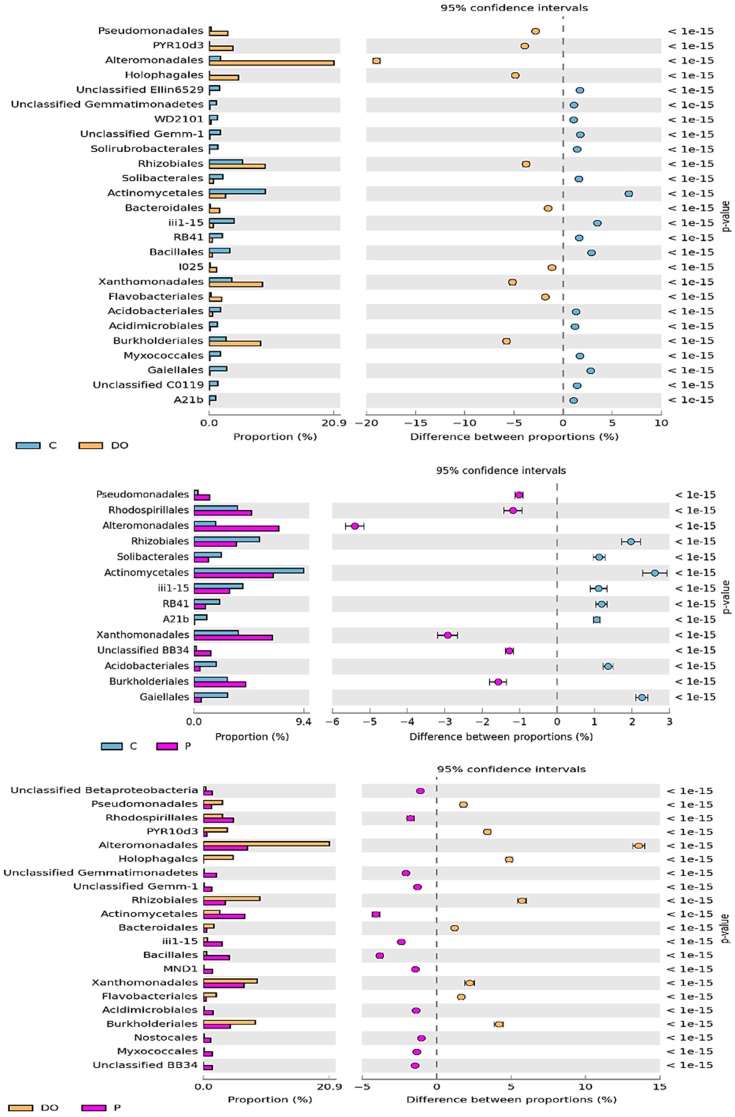
The relative abundance of dominant orders of bacteria in the soil with difference between proportions ≥1%. C—uncontaminated soil, DO—soil contaminated with diesel oil, P—soil contaminated with unleaded petroleum.

Differences in effects of the petroleum products were also noticeable at the order level (Figure 4). OTU abundance in the order rank was affected to a greater extent by DO than by P. Soil pollution with DO evoked the greatest changes in *Alteromonadales* classified to the *Gammaproteobacteria* class as it increased its OTU number by 19.0%, compared to an increase by 5.4% caused by soil exposure to P. A comparative analysis of the effects of both petroleum products allowed concluding that DO had a more beneficial effect on OTU abundance of *Rhizobiales* (class: *Alphaproteobacteria*), *Xanthomonadales* (class: *Gammaproteobacteria*), and *Burkholderiales* (class: *Betaproteobacteria*), but a negative effect on *Actinomycetales* (class: *Actinobacteria*).

**Figure 5:**
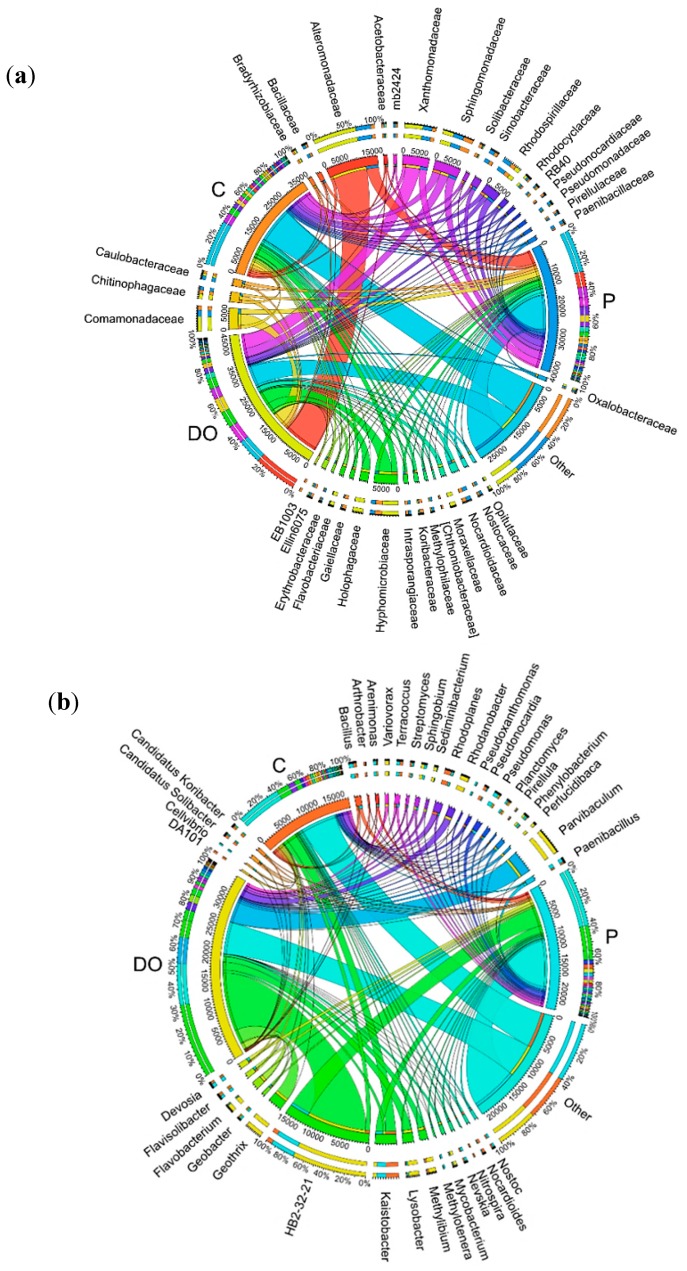
Abundance of bacterial communities at the family level (**a**) and genus level (**b**) in uncontaminated soil (C), soil contaminated with diesel oil (DO) and soil contaminated with unleaded petroleum (P). Abundances <1% are gathered into the category “other”.

Differences in OTU abundance in particular pots were also observed at the family level (Figure 5a). In the unpolluted soil, the highest number of OTUs was determined for the families: *Sphingomonadaceae* (7.4%), *Hyphomicrobiaceae* (5.3%), *Rhodospirillaceae* (4.0%), and *Xanthomonadaceae* (3.1%). In the DO-polluted soil, the order of families acc. to OTUs number was as follows: *Alteromonadaceae* (24.1%), *Xanthomonadaceae* (10.2%), *Comamonadaceae* (8.1%), and *Sphingomonadaceae* (6.1%), whereas in the P-polluted soil, it was: *Alteromonadaceae* (10.0%), *Sphingomonadaceae* (6.8%), *Xanthomonadaceae* (6.0%), *Rhodospirillaceae* (5.7%), and *Comamonadaceae* (4.3%). 

Diesel oil and unleaded petroleum also disturbed the soil microbiome at the genus level (Figure 5b), which was indicated by the preponderance of the genus *Kaistobacter* in the control soil, and of HB2-32-21 in the soil polluted with DO and P. 

**Figure 6:**
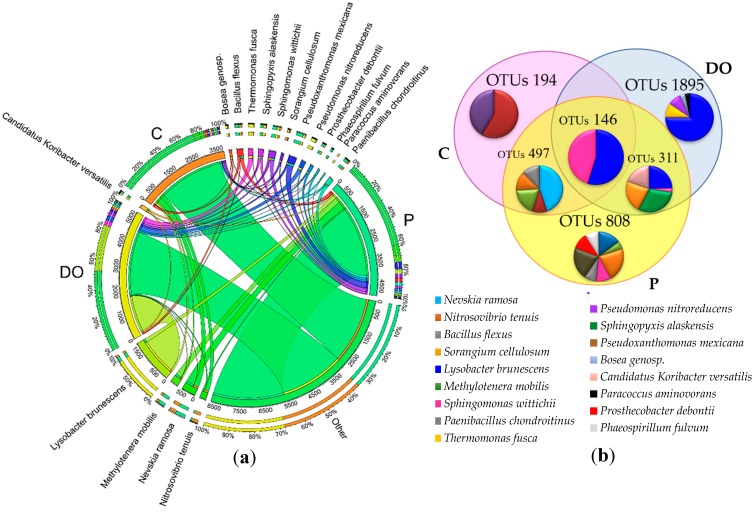
Abundance of bacterial communities at the species level (**a**) in uncontaminated soil (C), soil contaminated with diesel oil (DO) and soil contaminated with unleaded petroleum (P). Abundances <1% are gathered into the category “other”; (**b**) diagram of common OTU readings in uncontaminated soil (C), soil contaminated with diesel oil (DO) and soil contaminated with unleaded petroleum (P).

Considering the OTU numbers of individual bacterial species (OTUs higher than 1%), it is noteworthy that a higher number of species were classified in the soil polluted with petroleum products than in the control soil. The prevailing species in the unpolluted soil and in the soil polluted with P were *Nevskia ramosa*, which accounted for 5.9 and 7.0% respectively, whereas *Lysobacter brunescens* prevailed in the soil polluted with DO (29.9%) (Figure 6a). In total, 4951 OTUs were classified to the species. However, 146 OTUs were common for all soil types were examined (Figure 6b). In addition, 194 OTUs were typical only of the control soil, 1895 OTUs only for the DO-polluted soil, and 808 OTUs only for the P-polluted soil. 

To recapitulate the above considerations about the effect of petroleum products on soil health, it may be concluded that diesel oil caused greater changes in the soil microbiome than the unleaded petroleum. This was corroborated by the Shannon and Simpson (Table 1) indices computed based on OTU abundance, whose values were the lowest in the soil polluted with DO.

### 3.2. Activity of Soil Enzymes 

**Figure 7:**
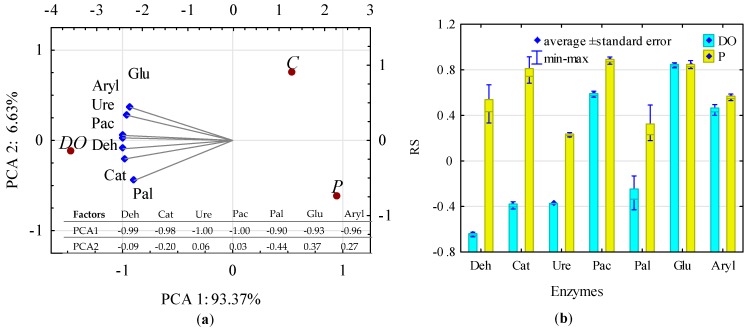
Activity of soil enzymes (**a**) presented with the PCA method; (**b**) enzyme resistance indices (RS) to soil contamination with diesel oil (DO) and unleaded petroleum (P). Deh—dehydrogenases; Cat—catalase, Ure—urease; Pac—acid phosphatase; Pal—alkaline phosphatase; Glu—β-glucosidase; Aryl—arylsulfatase. Homogeneous groups denoted with letters (a,b) were calculated separately for each of enzyme.

The activity of soil enzymes was significantly negatively correlated with the first principal component (PCA1) (Figure 7a). DO effect on soil enzymes was similar to its effect on microorganisms—it significantly stimulated their activity. Greater activity enhancement was observed in the case of oxidoreductases than in the case of hydrolases. The impact of P on soil enzymes was significantly lesser and explicitly negative in the case of urease (activity suppression by 63%), dehydrogenases (activity suppression by 36%), and arylsulfatase (activity suppression by 28%). The change in the biochemical properties of soil due to its pollution with petroleum products was reflected in the resistance (RS index) of individual enzymes to the effects of DO and P (Figure 7b). Enzymes resistance to diesel oil may be ordered as follows (in a descending order from the most to the least resistant ones): Glu > Pac > Aryl > Pal > Ure > Cat > Deh, while to unleaded petroleum—as follows: Pac > Glu > Cat > Aryl > Deh > Pal > Ure. 

### 3.3. Physicochemical Properties of Soil 

Apart from the direct impact on the microbiome of soil, the petroleum products affected its physicochemical properties (Table 2). Contrary to unleaded petroleum, soil pollution with diesel oil increased organic carbon content in the soil. In addition, both petroleum products contributed to a decrease in the total exchangeable base cations, soil saturation with bases, and sorption complex capacity. 

### 3.4. Degradation of Hydrocarbons 

The rate of degradation of individual hydrocarbons contained in diesel oil and petroleum varied (Table 3). In the case of both studied petroleum products, the fastest degradation was observed for ethylbenzene; m-, p-, and o-xylenes; toluene, and 2-ring PAHs. Degradation of the remaining pollutants was significantly affected by pollutant type. After 105 days, the soil exposed to the pressure of petroleum contained by 99.9% less benzines (C_6_-C_12_), by 98.1% less benzene, by 81.3% less mineral oil (C_12_-C_35_), by 97.6% less 3-ring PAHs, by 80.3% less 4-ring PAHs, by 76.8% less 5-ring PAHs, and by 60.6% less 6-ring PAHs, whereas contents of the respective compounds in the soil polluted with petroleum were lower by: 89.1%, 75.0%, 63.0%, 87.6%, 45.2%, 25.0%, and 13.3%. 

### 3.5. Response of Elymus Elongatus 

**Figure 8:**
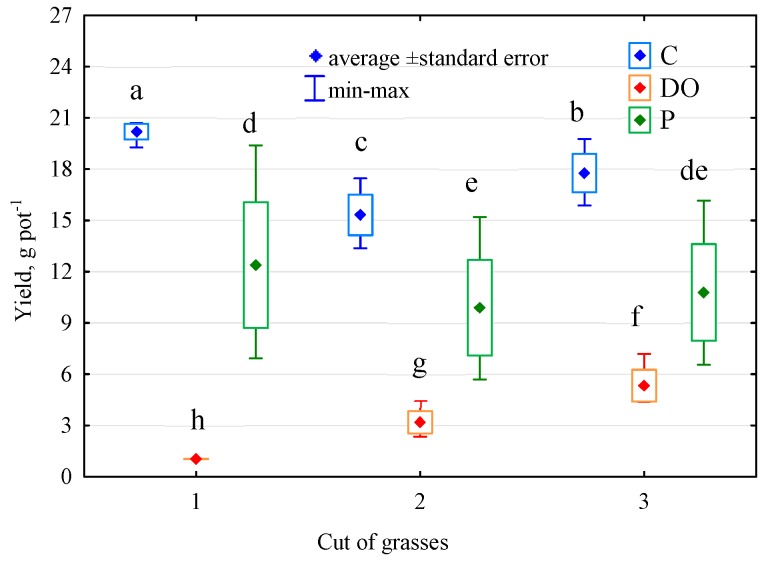
Yields of *Elymus elongatus* in cuts number 1, 2 and 3 (g DM pot^−1^). Homogeneous groups denoted with letters (a–h) were calculated for yield.

Soil pollution with petroleum products disturbed the growth and development of *Elymus elongatus* in the entire growing season (Figure 8). This was confirmed by its yield obtained in particular swaths. A stronger toxic effect was observed for DO than for P, as DO decreased the total yield of *Elymus elongatus* by as much as 82%, whereas P by 38%. The RS value of *Elymus elongatus* was lower under soil exposure to DO than to P, but the adverse effect of both products on the test plant sustained throughout the study period, which was indicated by low RL values (Figure 8, Table 4).

## 4. Discussion

### 4.1. Counts and Diversity of Microorganisms in the Soil 

The pollution of soil with petroleum products leads to their successive degradation, which results in productivity decrease [49], and to soil microbiome changes [4,50,51]. Also, in the present study were the DO and P observed to destabilize the microbiological life of soil. Namely, DO significantly stimulated the proliferation of all microorganisms, whereas P caused no changes in the population numbers of organotrophic bacteria and fungi. Earlier investigations [6,52,53,54,55] have indicated that diesel oil usually causes greater changes in the proliferation of soil microorganisms than petroleum. DO decreased values of the ecophysiological diversity index (EP) of organotrophic bacteria, Actinobacteria, and fungi, whereas P decreased EP of Actinobacteria. This is due to the succession of microorganisms utilizing various chemical compounds of DO and P [56,57], which is accompanied by changes in soil properties, i.e., disruption of trophic and aerobic conditions, and excess of active forms of organic carbon [58,59,60,61]. 

The results of the present study concerning the genetic diversity of bacteria proved *Proteobacteria* to be the prevailing *Phylum* in the unpolluted soil. After its contamination with petroleum products, higher OTU numbers were demonstrated for *Actinobacteria* and *Acidobacteria*. According to Gałązka et al. [50], soil samples collected 0.5 to 3 m away from oil wells were colonized mainly by *Alphaproteobacteria*, *Betaproteobacteria*, and *Gammaproteobacteria,* which were strongly correlated with the biological activity of these soils. The prevailing classes included also *Actinobacteria* and *Acidobacteria*. The high counts of bacteria classified to *Proteobacteria, Bacteroidetes*, and *Actinobacteria* in the soils polluted with petroleum products were also confirmed by results of investigations conducted by Yan et al. [20], Hou et al. [62], and Jung et al. [63]. These taxa, potentially capable of degrading alkanes, being major components of diesel oil, diminished the diversity of microorganisms [63]. Soil pollution with petroleum substances induces far-reaching changes noticeable also at the lower taxonomic levels [50]. In the present study, diesel oil increased the OTU abundance of *Alteromonadaceae, Xanthomonadaceae, Comamonadaceae,* and *Sphingomonadaceae*, whereas unleaded petroleum of: *Alteromonadaceae, Sphingomonadaceae, Xanthomonadaceae, Rhodospirillaceae,* and *Comamonadaceae*. Gałązka et al. [50] demonstrated *Bradyrhizobiaceae*, *Rhizobiaceae*, *Rhodobacteraceae*, *Acetobacteraceae*, *Hyphomicrobiaceae*, and *Sphingomonadaceae* classified to *Alphaproteobacteria* to be the prevailing species of the soil located directly near an oil well. In turn, Feng et al. [64] and Afzal et al. [65] reported that the soils polluted with petroleum products were colonized mainly by *Pseudomonaceae*, *Burkholderiaceae*, *Bacillaceae*, and *Enterobacteriaceae*. The petroleum products modify the genus and species abundance of microorganisms [20]. In the soils polluted with DO and P, the greatest OTU abundance was found for HB2-32-21. According to Mukherjee et al. [66], these bacteria are effective in the remediation of areas polluted with petroleum products, and Czarny et al. [67] claims they adapt very fast to conditions occurring in such soils. This may be due to the availability of an alternative source of carbon [63].

### 4.2. Activity of Soil Enzymes 

Petroleum products which have pervaded the soil cause changes not only in the diversity of microorganisms but also in the enzymatic activity of soil [68]. These changes lead to modifications of protein conformation associated with membranes and of proton pumps [69]. The enzymatic activity of soil is a reliable indicator of its health status, because changes in the soil are reflected faster in its enzymatic activity than in its other properties [56,57]. Dehydrogenases, β-glucosidase, urease, acidic and alkaline phosphomonoesterase, and arylsulfatase are claimed to be common indicators of C, N, P, and S metabolism [26,70,71]. In the present study, the response of soil enzymes to diesel oil was coincident with microorganism response to these compounds, i.e., diesel oil increased counts of microorganisms and also activity of soil enzymes. This is logical, because microorganisms represent the major source of soil enzymes [56,57,70]. In particular, almost a 6-fold enhancement of the activity of dehydrogenases in the soil exposed to the pressure of diesel oil and its 0.6-fold suppression in the soil polluted with unleaded petroleum compared to the control soil may be due to the fact that dehydrogenases are intracellular enzymes which are strongly associated with the number and biomass of microorganisms [68]. Opposite effects of both tested substances on the enzymatic activity are attributable to significant differences in their physical and chemical properties [72]. 

### 4.3. Degradation of Hydrocarbons 

A mixture of hydrocarbons, like that in the petroleum products, is especially hazardous to soil health [51]. Hence, these compounds should be removed from polluted soils as fast as possible. The rate of PAHs degradation in the soil is largely affected by the type of petroleum pollutant [6,7,73] and by plant species used for phytoremediation [16,22,74]. In the present study, the degradation of hydrocarbons was significantly affected by the petroleum product. After 105 days, significantly more PAHs were degraded in the soil polluted with unleaded petroleum than with diesel oil, which is consistent with *Elymus elongatus* response to these substances. This is due to the greater resistance of these plants to P than to DO and to a better developed root system of tall wheatgrass on the soil exposed to petroleum, and by this means offering more favorable water-air conditions for more rapid degradation of PAHs [29]. 

Both, results from our study and literature data [10,75] indicate that some groups of organic compounds being constituents of petroleum products exert long-lasting effects on the soil environment. According to Xu and Lu [76], from 26% to 61% of petroleum hydrocarbons may be degraded in barely 90 days. A pot experiment conducted by Liu et al. [74] with 14 species of grasses and ornamental plants has demonstrated that PHAs degradation rate ranges from 37% do 49%. In turn, the present study proves that degradation of PAHs within 105 days may range from 13% to 99% depending on their chemical properties. Likewise, in the study conducted by García-Sánchez et al. [75], the fastest degradation rate was observed for the 2–3-ring PAHs, and the slowest one for the 5–6-ring ones. This is probably due to the fact that PAHs with a simpler chemical structure are easier sources of carbon and electron donors for autochthonous microorganisms of soil. Nanekar et al. [77] emphasized also that 2- and 3-ring hydrocarbons are more susceptible to evaporation and photoxidation than these with a higher number of rings. 

### 4.4. Physicochemical Properties of Soil

The effect of petroleum products on the physicochemical properties of soil is affected, to a great extent, by soil quality [78], petroleum product type [79], and pollution magnitude [80,81]. In the present study, the sum of exchangeable cations, exchangeable capacity, and soil saturation with base cations were significantly diminished by DO and P. This effect was due to the negative impact of these products on, i.a., air-water properties [73,82,83]. According to Rasheed et al. [84], soil pollution with DO and P may cause the loss of their elasticity and viscosity. In turn, Prasanna and Manoharan [85] ascribe this unfavorable effect to a greater pool of physical and chemical parameters changing in soil upon its pollution with these products. This is also reflected in the modified microbiological and biochemical properties of soil. 

### 4.5. Plants Response 

Petroleum products disturb the growth and development of plants [16,17,73]. Nevertheless, plant response to soil pollution with these products depends on pollutant type [73,86] and plant species [5,26,87]. The proper choice of plant species for the phytoremediation process is of the outmost significance [15,17]. A prerequisite for effective degradation of organic pollutants is a well-developed root system of plants [16,21,23], because it prevents organic pollutants migration across the environment. One of such plants meeting these criteria is *Elymus elongatus* which was used in our study. Tall wheatgrass was more resistant to soil pollution with unleaded petroleum than with diesel oil, which was indicated by indices of *Elymus elongatus* resistance to soil pollution with these petroleum products. 

The results of the present study prove that DO exerted a stronger toxic effect on *Elymus elongatus* than P did. Also, investigations conducted by other authors [5,6,73,86,88] have demonstrated that petroleum products negatively affect the growth and development of plants such as *Zea mays, Avena sativa, Lupinus luteus, Vulpia myuros,* and *Phalaris arundinacea.* Although in our study the negative impact of DO diminished with time (since soil contamination) and that of P remained stable throughout the growing season, the soil polluted with these substances was characterized by a low capability of returning to the equilibrium state. The mean value of RL index computed based on *Elymus elongatus* yield in the soil exposed to the pressure of diesel oil was 0.213 and that computed in the soil polluted with unleaded petroleum was 0.056.

## 5. Conclusions

The correct risk assessment of soils polluted with petroleum products is indispensable to identifying possibilities for the management of such ecosystems. Hence, the accurate evaluation of changes proceeding in real time in soils under the pressure of petroleum substances is of great significance. The analysis of microbiological, biochemical, physicochemical, and chemical indices of soil coupled with the *Elymus elongatus* response allowed for the complex assessment of changes in the microbiome of soil exposed to the effects of BP diesel oil and unleaded petroleum with Active technology. Soil pollution with these petroleum products upset the soil metabolic profile, whereas their hydrocarbons were relatively resistant to biodegradation. The study results enable concluding that the quality of soil polluted with these products may be improved through adjusting plant species used for phytoremediation to pollutant type. By showing greater resistance to the effects of petroleum than to these of diesel oil, *Elymus elongatus* contributed to dynamic degradation of PAHs from the soil. Soil contamination with diesel oil (DO) and unleaded petroleum (P) in the amount of 7 cm^3^ kg^−1^ soil significantly impaired the growth and development of *Elymus elongatus* throughout its growing season. A significantly greater decrease in its biomass was caused by DO than by P. The usability of *Elymus elongatus* for the remediation of soil contaminated with the tested pollutants is unquestionable in the case of soil contamination with unleaded petroleum, and relatively low in the case of soil contaminated with diesel oil, which was indicated by the significantly higher degradation of hydrocarbons in the soil polluted with P than with DO. The petroleum products also disturbed the stability of the soil microbiome. Under conditions of the conducted experiment, greater negative effects were caused by DO than by P. This is a feedback between the effects of these pollutants on soil microorganisms and on the growth and development of *Elymus elongatus.*

## Figures and Tables

**Table 1:** Shannon and Simpson indices calculated from the abundance of operational taxonomic unit OTU.

Object	Phylum	Class	Order	Family	Genus	Species
Shannon Index						
C	2.13 ^a^	3.34 ^a^	3.87 ^a^	4.21 ^a^	4.13 ^a^	2.75 ^b^
DO	1.23 ^c^	2.26 ^c^	3.06 ^c^	3.04 ^c^	2.82 ^b^	1.68 ^c^
P	2.00 ^b^	3.16 ^b^	3.75 ^b^	3.99 ^b^	4.06 ^a^	2.87 ^a^
Simpson Index						
C	0.81 ^a^	0.94 ^a^	0.96 ^a^	0.98 ^a^	0.97 ^a^	0.90 ^a^
DO	0.48 ^c^	0.79 ^b^	0.91 ^a^	0.90 ^b^	0.84 ^b^	0.61 ^b^
P	0.74 ^b^	0.92 ^a^	0.96 ^b^	0.97 ^a^	0.95 ^a^	0.92 ^a^

C—uncontaminated soil, DO—soil contaminated with diesel oil, P—soil contaminated with unleaded petroleum. Homogeneous groups denoted with letters (a, b, c) were calculated separately for each of taxon.

**Table 2:** Physicochemical properties of uncontaminated soil (C) and polluted with diesel oil (DO) and unleaded petroleum (P).

Object	C_org_	EBC	HAC	CEC	BS	pH_KCl_
	g kg^−1^	mmol(+) kg^−1^			%	
C	26.9 ^b^	69.3 ^a^	11.3 ^b^	80.6 ^a^	86.0 ^a^	6.7 ^c^
DO	28.3 ^a^	62.0 ^b^	11.3 ^b^	70.3 ^b^	84.6 ^b^	7.0 ^a^
P	26.3 ^b^	58.7 ^c^	12.0 ^a^	70.7 ^b^	83.0 ^c^	6.9 ^a,b^

C—uncontaminated soil, DO—soil contaminated with diesel oil, P—soil contaminated with unleaded petroleum. C_org_—organic carbon content; EBC—exchangeable base cations; HAC—hydrolytic acidity; CEC—cation exchange capacity; BS—base saturation. Homogeneous groups denoted with letters (a, b, c) were calculated separately for each of physicochemical properties.

**Table 3:** Degradation of hydrocarbons in soil contaminated with diesel oil (DO) and unleaded petroleum (P). %.

**Object**	**C_6_–C_12_**	**C_12_–C_35_**	**Ben**	**EtB**	**Tol**	**Xyl**	**Sty**	**∑ BTEX**	**Nap**	**Ant**
DO	89.1 ^b^	63.0 ^b^	75.0 ^b^	99.4 ^a^	98.6 ^b^	99.6 ^a^	0.0 ^b^	99.3 ^a^	99.2 ^b^	87.6 ^b^
P	99.9 ^a^	81.3 ^a^	98.1 ^a^	99.9 ^a^	100.0 ^a^	99.9 ^a^	96.7 ^a^	99.9 ^a^	99.8 ^a^	66.6 ^a^
**Object**	**Chr**	**BaA**	**DahA**	**BaP**	**BbF**	**BkF**	**BghiP**	**IP**	**9PAHs**	**10PAHs**
DO	47.8 ^b^	37.5 ^b^	0.0 ^b^	44.4 ^b^	25.0 ^b^	20.0 ^b^	0.0 ^b^	25.0 ^b^	95.3 ^b^	95.8 ^a^
P	82.4 ^a^	77.6 ^a^	50.0 ^a^	76.5 ^a^	77.6 ^a^	80.6 ^a^	97.3 ^a^	97.9 ^a^	99.0 ^a^	95.6 ^a^

C_6_–C_12_—gasoline fractions _;_ C_12_–C_35_—mineral oil; Ben—benzene; EtB—ethylbenzene; Tol—toluene; X—xylene; Sty—styrene; ∑ BTEX—∑ volatile hydrocarbons BTEX; Nap—naphthalene; Ant—anthracene; Chr—chrysene; BaA—benzo[a]anthracene; DahA—dibenz(a,h)anthracene; BaP—benzo(a)pyrene; BbF—benzo[b]fluoranthene; BkF—benzo(k)fluoranthene; BghiP—benzo(ghi)perylene; IP—indeno(1,2,3-cd)pyrene; 9 PAHs—∑ 9 polycyclic aromatic hydrocarbons; 10 PAHs—∑ 10 polycyclic aromatic hydrocarbons. C—uncontaminated soil, DO—soil contaminated with diesel oil, P—soil contaminated with unleaded petroleum. Homogeneous groups denoted with letters (a, b) were calculated separately for each of hydrocarbons.

**Table 4:** *Elymus elongatus* resistance index (RS) and adaptation (RL) index to contamination with diesel oil (DO) and unleaded petroleum (P).

Object		RS		RL
	Cut 1	Cut 2	Cut 3	
DO	0.026 ^c^	0.116 ^b,c^	0.176 ^b^	0.213 ^a^
P	0.442 ^a^	0.476 ^a^	0.436 ^a^	0.056 ^b^

Homogeneous groups denoted with letters (a, b, c) were calculated separately for RS and RL.
